# Enzymatic Synthesis of Novel Vitexin Glucosides

**DOI:** 10.3390/molecules26206274

**Published:** 2021-10-16

**Authors:** Jiumn-Yih Wu, Tzi-Yuan Wang, Hsiou-Yu Ding, Yun-Rong Zhang, Shu-Yuan Lin, Te-Sheng Chang

**Affiliations:** 1 Department of Food Science, National Quemoy University, Kinmen County 892, Taiwan; wujy@nqu.edu.tw; 2 Biodiversity Research Center, Academia Sinica, Taipei 11529, Taiwan; tziyuan@gmail.com; 3 Department of Cosmetic Science, Chia Nan University of Pharmacy and Science, Tainan 71710, Taiwan; ding8896@gmail.com; 4 Department of Biological Sciences and Technology, National University of Tainan, Tainan 70005, Taiwan; S10758011@gm2.nutn.edu.tw (Y.-R.Z.); s10758010@gm2.nutn.edu.tw (S.-Y.L.)

**Keywords:** glycoside hydrolase, glycosyltransferase, glycosylation, vitexin, glucoside

## Abstract

Vitexin is a *C*-glucoside flavone that exhibits a wide range of pharmaceutical activities. However, the poor solubility of vitexin limits its applications. To resolve this limitation, two glycoside hydrolases (GHs) and four glycosyltransferases (GTs) were assayed for glycosylation activity toward vitexin. The results showed that *Bt*GT_16345 from the *Bacillus thuringiensis* GA A07 strain possessed the highest glycosylation activity, catalyzing the conversion of vitexin into new compounds, vitexin-4′-*O*-*β*-glucoside (**1**) and vitexin-5-*O*-*β*-glucoside (**2**), which showed greater aqueous solubility than vitexin. To our knowledge, this is the first report of vitexin glycosylation. Based on the multiple bioactivities of vitexin, the two highly soluble vitexin derivatives might have high potential for pharmacological usage in the future.

## 1. Introduction

**Figure 1:**
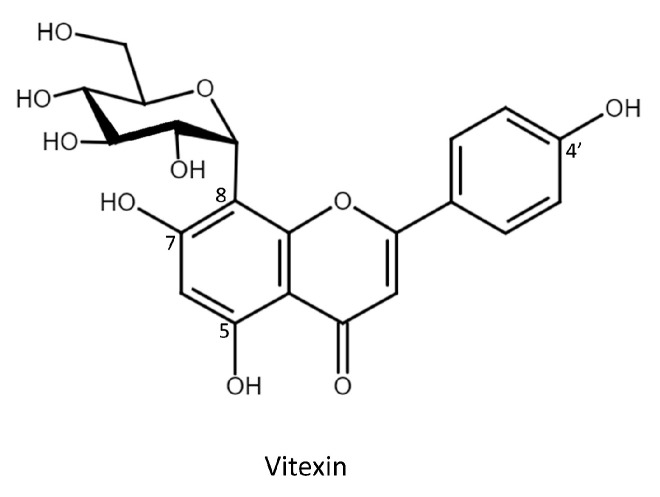
Chemical structure of vitexin.

Vitexin (apigenin-8-*C*-glucoside, Figure 1), referred to as ‘mujingsu′ in Chinese, is a cancer therapy compound found in many traditional Chinese medicines. It is a *C*-glycosylated flavone and is, thus, largely resistant to molecular transformation. Vitexin is found in various plants, such as mung bean, chasteberry, buckwheat, hawthorn, passion flowers, pearl millet, and bamboo. These agricultural plants have also been used in traditional medicine for 3000 years [1]. Vitexin has recently received increased attention due to its wide range of pharmacological activities, which include anti-oxidant [2], anti-cancer [3], anti-diabetes [4], and neuroprotective [5] effects. Its therapeutic effects are linked to several bodily systems, such as the central nervous system, cardiovascular system, and endocrine system. Thus, vitexin is a valuable phytochemical and has high potential for pharmaceutical usage. However, vitexin has poor aqueous solubility [6], which leads to poor bioavailability and low absorption in the gastrointestinal tract (approximately 5%) [7]. Glycosylation is a promising strategy, and glycosylated derivatives of such compounds have been shown to have improved aqueous solubility [8,9]. In addition, glycosylation has been shown to enhance the oral bioavailability of the original molecules [10,11,12]. Therefore, it is vital to determine whether glycosylation can improve the aqueous solubility of vitexin.

Molecules can be modified using chemical and biological (enzymatic) methods. In terms of glycosylation, the multiple hydroxyl groups in the sugar moiety and the non-regioselectivity of the chemical reaction make it difficult to achieve glycosylation using chemical methods. In contrast, enzymatic glycosylation has several advantages, such as mild reaction conditions, high regioselectivity and stereoselectivity, and fewer side reactions. Therefore, enzymatic glycosylation of molecules is easier than chemical glycosylation [13]. Given the dearth of studies on the glycosylation of vitexin and the need to further examine vitexin, the enzymatic glycosylation of vitexin was investigated as a case study.

Two types of enzymes have been shown to glycosylate such molecules: Leloir glycosyltransferases (GTs) [14,15] and some glycoside hydrolases (GHs) [16,17]. According to the carbohydrate-activating enzyme (CAZy) database, 114 GT families and 117 GH families have been discovered to date. GTs catalyze glycosylation by transferring the sugar moiety from a donor molecule, such as uridine–diphosphate glucose (UDP-G), to an acceptor molecule. GT family 1 (GT1) members catalyze the glycosylation of small molecules, such as phenolics. In addition to hydrolytic activity, several GHs in GH family 13 (GH13) and GH family 68 (GH68) have also shown glycosylation ability by transferring the sugar moiety from donor to acceptor molecules [16].

In this study, two known GHs and four known GTs were selected to biotransform vitexin via glycosylation. The four *Bacillus* GTs were found to be able to glycosylate vitexin. The new vitexin glucosides were further isolated, and their chemical structures and solubilities were determined.

## 2. Results and Discussion

### 2.1. Glycosylation of Vitexin by GHs and GTs

To study the glycosylation of vitexin (Figure 1), one GT28 (*Bt*GT_16345), three GT1 (*Bs*GT110, *Bs*UGT398, and *Bs*UGT489), and two GH13 (*Dg*AS and *Pg*MA) enzymes were selected to catalyze the glycosylation (Table 1). The six selected enzymes have proven glycosylation activity toward flavonoids or triterpenoids [18,19,20,21,22]. Each set of reaction conditions was the same as that used in previous studies. After a 24 h reaction, the glycosylated mixture was mixed with an equal volume of methanol and analyzed using HPLC. The results showed that *Bt*GT_16345 from *Bacillus thuringiensis* GA A07 strain glycosylated vitexin to produce compounds (**1**) and (**2**) with yields of 17.5% and 18.6%, respectively (Supplementary Materials Figure S1 and Table 1). Second, the three GT1 enzymes from *B. subtilis* also glycosylated vitexin, but only produced compound (**1**) at lower yields (5–13%). The two GH13 enzymes did not catalyze the glycosylation of vitexin. The results indicate that the glycosylation of vitexin is more challenging for GH enzymes. Nevertheless, these results reveal that *Bacillus* GTs can glycosylate vitexin to produce vitexin glucosides, which could be applied in pharmaceuticals.

### 2.2. Isolation and Identification of Vitexin Glycosylation Products Produced by BtGT_16345

**Figure 2:**
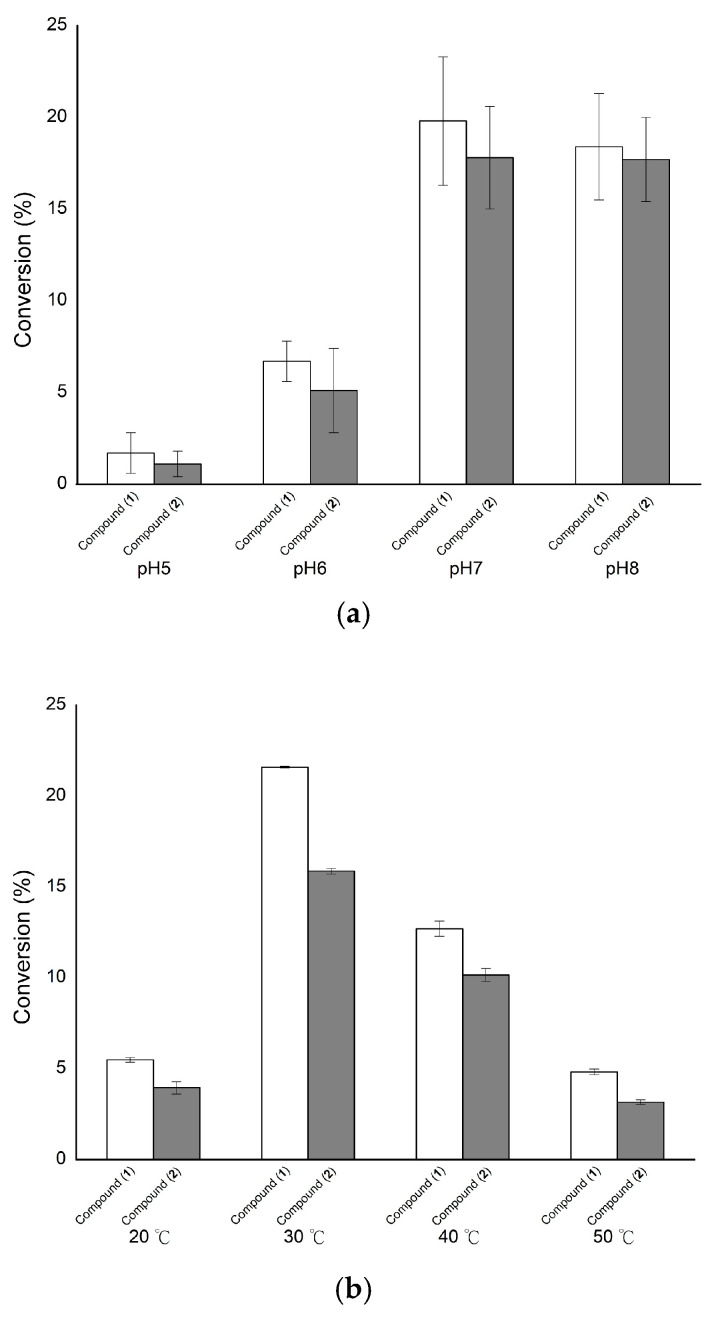

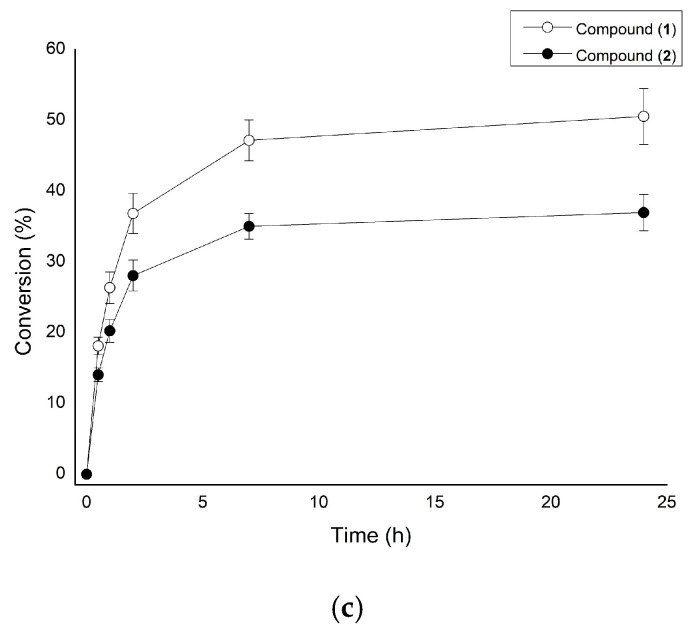
Effects of pH (**a**), temperature (**b**), and reaction time (**c**) on the glycosylation of vitexin by *Bt*GT_16345. The standard condition was set to 25 µg/mL *Bt*GT_16345, 1 mg/mL vitexin, 10 mM uridine diphosphate–glucose (UDP-G), 10 mM MgCl_2_, and 50 mM phosphate buffer (pH 7) at 30 °C for 30 min. To determine suitable reaction conditions, different pH values, temperatures, and reaction times were tested. After incubation, the biotransformation products were analyzed, using high-performance liquid chromatography (HPLC). The detailed reaction conditions and the HPLC procedure are described in the Materials and Methods section.

Since the glycosylation yields were highest with *Bt*GT_16345, the glycosylated mixture resulting from the use of this enzyme was selected for scale up and purification. The biotransformation of vitexin by *Bt*GT_16345 was first optimized by testing at different pH (pH 5–8), temperatures (20–50 °C) and reaction times (0–24 h). The results showed that the optimal conditions were pH 7 at 30 °C for 24 h (Figure 2). The biotransformation was then scaled up to a 100 mL reaction, with 100 mg of vitexin as the substrate. The reaction mixture was mixed with an equal volume of methanol and purified with a preparative HPLC system. The eluate corresponding to the metabolite peak in the analytical HPLC was collected, condensed under a vacuum, and then crystallized by lyophilization. Finally, the yields of compound (**1**, 14.2 mg) and compound (**2**, 15.5 mg) were 14.2 and 15.5%, respectively.

The chemical structures of compounds (**1**) and (**2**) were revealed, using mass and nuclear magnetic resonance (NMR) spectral analysis. The mass analyses of compounds (**1**) and (**2**) showed an identical [M − H]^−^ ion peak at *m*/*z*: 593.3 in the electrospray ionization mass (ESI-MS) spectrum, corresponding to the molecular formula C_27_H_30_O_15_ and the vitexin glucoside. Furthermore, the mass–mass analysis of the [M − H]^−^ ion peak at *m*/*z*: 593.3 showed another [M − H]^−^ ion peak at *m*/*z*: 431.3 corresponding to vitexin (molecular weight of 432 Da) (Figures S2 and S3). The results strongly implied that both compounds (**1**) and (**2**) were vitexin glucosides, and NMR spectroscopic analysis further defined their structures.

The ^1^H- and ^13^C-NMR spectra, including distortionless enhancement by polarization transfer (DEPT), heteronuclear single quantum coherence (HSQC), heteronuclear multiple bond connectivity (HMBC), correlation spectroscopy (COSY), and nuclear Overhauser effect spectroscopy (NOESY) spectra, were obtained, and the ^1^H- and ^13^C-NMR signal assignments were conducted accordingly. The ^1^H-NMR spectrum of compound (**1**) exhibited a flavonoid pattern with four aromatic signals: an AA′BB′ system at δ H 6.16 (2H, d, *J* = 8.8 Hz, H-3′/H-5′) and δ H 8.14 (2H, d, *J* = 8.8 Hz, H-2′/H-6′), and two additional aromatic signals at δ H 6.29 (1H, s, H-6) and δ H 6.90 (1H, s, H-3). The carbon atoms of the aforementioned protons were found to resonate at 128.7 ppm (C-2′/C-6′), 116.6 ppm (C-3′ /C-5′), 103.5 ppm (C-3), and 98.2 ppm (C-6), respectively. The ^1^H-NMR signals reflected the *C*-glucosyl (δ H: 3.24 (H-5′′), 3.27 (H-3′′), 3.41 (H-4′′), 3.56 (H-6′′a), 3.78 (H-6′′b), 3.81 (H-2′′), 4.69 (H-1′′)) and *O*-glucosyl (δ H: 3.19 (H-4′′′), 3.28 (H-2′′′), 3.31 (H-3′′′), 3.37 (H-5′′′), 3.48 (H-6′′′a), 3.69 (H-6′′′b), 5.03 (H-1′′′)) of compound (**1**).

Finally, characteristic ^1^H- and ^13^C-NMR sugar signals were assigned to *C*-glucosyl and *O*-glucosyl moieties in 1D- and 2D-NMR experiments. The glycosidic linkage of the *C*-glucosyl unit on the flavonoid A-ring was confirmed, using HMBC and NOESY correlations occurring between H1′′/C7, C8, C9, and the NOESY correlation between H1′′/H2′′. The grafting of the *O*-glucosyl unit onto the flavonoid *C*-ring was revealed by the presence of HMBC correlations between H1′′′/C4′ and the NOESY correlation between H1′′′/H3′ (5′). The doublet signal at δH H-1′′ (d, 1H, *J* = 9.8 Hz) and H-1′′′ 5.03 (d, 1H, *J* = 7.0 Hz) with the corresponding carbon atom at C-1′′ (73.4 ppm) and C-1′′′ (100.0 ppm) was assigned to the anomeric proton and indicated a *C*-*β*-configuration and an *O*-*β*-configuration, respectively. Hence, compound (**1**) was assigned as vitexin-4′-*O*-*β*-glucoside (**1**). The NMR spectroscopic data are shown in Figures S4–S10.

**Figure 3:**
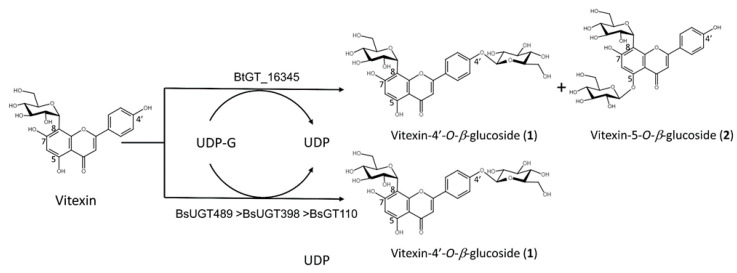
Biotransformation process of vitexin by *Bacillus* GTs.

According to ^1^H- and ^13^C-NMR data, compound (**2**) was structurally similar to vitexin-4′-*O*-*β*-glucoside (**1**), described above. The aromatic signals at δ H 6.61 (1H, s, H-6) and δH 6.88 (1H, s, H-3) with their corresponding carbons resonating at 98.5 ppm (C-6) and 102.6 ppm (C-3) were identified. The signals were attributed to two glucose moieties at the *C*-glucosyl (δ H: 3.29 (H-5′′), 3.39 (H-3′′), 3.40 (H-4′′), 3.52 (H-6′′a), 3.72 (H-2′′), 3.78 (H-6′′b), 4.87 (H-1′′)) and *O*-glucosyl (δ H: 3.21 (H-4′′′), 3.31 (H-3′′′), 3.35 (H-2′′′), 3.42 (H-5′′′), 3.49 (H-6′′′a), 3.71 (H-6′′′b), 4.96 (H-1′′′)) of compound (**2**). The anomeric signal of the *C*-*β*-glucoside and *O*-*β*-glucoside moieties resonated as a doublet at δH 4.87 (d, 1H, *J* = 9.8 Hz, H-1′′) and δH 4.96 (d, 1H, *J* = 7.7 Hz, H-1′′′) with the HSQC corresponding carbon atom at 73.3 ppm (C-1′′) and 101.2 ppm (C-1′′′), respectively. An ether linkage between the H-1′′ of glucose and the C-8 (4.87/107.2 ppm), H-1′′′, and C-5 (4.96/161.2 ppm) of compound (**2**) was proven, using the HMBC and NOESY (H-1′′′/H6) spectra. The structure of compound (**2**) was thus confirmed to be vitexin-5-*O*-*β*-glucoside (**2**). The NMR spectroscopic data are shown in Figures S11–S17. Figure 3 summarizes the biotransformation process of vitexin by the *Bacillus* GTs.

### 2.3. Characterization of Vitexin Glucosides

The overall aim of this work is to improve the aqueous solubility of vitexin through glycosylation to enable its clinical application. Given that the solubility of flavonoids is affected by the pH of the solvent, the aqueous solubility of vitexin and its two glycosylated derivatives was determined at different pH (Table 2). It was found that the solubility of vitexin and its glucosides is higher in alkaline solution. In addition, the aqueous solubility of vitexin-4′-*O*-*β*-glucoside and vitexin-5-*O*-*β*-glucoside was higher than that of vitexin.

It is known that flavonoid glycosides are more stable than their aglycones under intestinal conditions [23,24]. *C*-glycosidic flavonoids (e.g., vitexin) are also more resistant than *O*-glycosidic flavonoids to acidic and enzymatic hydrolysis and are thus more stable during oral absorption [1,7]. However, a recent study indicated that enzymatic *O*-glycosylated vitexin could be deglycosylated and reverted to vitexin under in vitro intestinal conditions [25] and in fecal fermentation [25,26]. *β*-Glucosidases play a key role in the deglycosylation of the flavonoid *β*-glucosides, such as the two vitexin glucosides. In the human body, cytosolic *β*-glucosidase (hCGB, 3.2.1.21) can hydrolyze *β*-glycosylated xenobiotics and various flavonoid *β*-glucosides [27]. It has also been found that the human colonic microbiota possesses *β*-glucosidases, which can deglycosylate plant flavonoid glucosides and xenobiotics, allowing these compounds to be absorbed by the colon [28,29]. These studies revealed that fecal microbes can metabolize and remove the sugars of *O*-glycosylated flavonoids, which serve as easily accessible energy sources in symbiosis. Therefore, the two highly soluble vitexin *O*-*β*-glucosides identified in the current study might be digested by intestinal microbes to produce vitexin during oral absorption. From this perspective, the two vitexin *O*-*β*-glycosides may have future pharmacological use as vitexin prodrugs.

Conversely, many bioactive compounds are glycosides, and the glycosidic residues are highly correlated to the bioactivity. Some glycosides possess specific bioactivity that cannot be simply derived from the activity of the corresponding aglycone. Although it is nearly impossible to define the general pattern of bioactivities between the glycosides and the corresponding aglycons, the roles of glycosidic residues in bioactivities have been discussed [30]. Herein, the two vitexin glucosides might possess different bioactivities to vitexin, and this will be studied in the future.

## 3. Materials and Methods

### 3.1. Enzymes and Chemicals

The recombinant GHs (amylosucrase from *Deinococcus geothermalis* (*Dg*AS) [18] and maltogenic amylase from *Parageobacillus galactosidasius* (*Pg*MA) [19]) and the recombinant *Bacillus* GTs (*Bs*GT110 [20], *Bs*UGT398, *Bs*UGT489 [21], and *Bt*GT_16345 [22]) were obtained and purified, according to previous studies. Vitexin was purchased from Baoji Herbest Bio-Tech (Xi-An, Shaanxi, China). UDP-G was obtained from Cayman Chemical (Ann Arbor, MI, USA). Maltodextrin (dextrose equivalent 4.0–7.0) was bought from Sigma (St. Louis, MO, USA). The other reagents and solvents used were commercially available.

### 3.2. Glycosylation of Vitexin by GTs and GHs

Glycosylation of vitexin by *Dg*AS was performed using sucrose as a sugar donor [18]. The reaction mixture contained 1 mg/mL vitexin diluted from a 20 mg/mL stock dissolved in dimethyl sulfoxide (DMSO), 25 µg/mL *Dg*AS, 50% (*w*/*v*) sucrose, and 50 mM phosphate buffer (PB) pH 7.0. The reaction was performed at 40 °C for 24 h.

Glycosylation of vitexin by *Pg*MA was performed, using maltodextrin as a sugar donor [19]. The reaction mixture contained 50% (*w*/*v*) maltodextrin, 5.6 µg/mL *Pg*MA, 1 mg/mL vitexin, and 50 mM PB (pH 7) and was incubated at 65 °C for 24 h.

The glycosylation of vitexin by *Bacillus* GTs was performed, using UDP-G as a sugar donor [20,21,22]. The biotransformation mixture containing 25 µg/mL purified recombinant GT enzymes, 1 mg/mL vitexin, 10 mM UDP-G, 10 mM MgCl_2_, and 50 mM PB at pH 6 (*Bs*GT110) or pH 7 (*Bt*GT_16345), or Tris buffer at pH 8 (*Bs*UGT398 and *Bs*UGT489) was incubated at 30 °C (*Bs*GT110 and *Bt*GT_16345) or 40 °C (*Bs*UGT398 and *Bs*UGT489) for 30 min. For optimal pH experiments, the reaction mixture containing 25 µg/mL *Bt*GT_16345, 1 mg/mL vitexin, 10 mM UDP-G, 10 mM MgCl_2_, and 50 mM of buffer at pH 5 (acetate buffer), pH 6–7 (PB), or pH 8 (Tris) was incubated at 30 °C for 30 min. For optimal temperature experiments, the same reaction mixture with 50 mM of PB at pH 7 was incubated at different temperatures for 30 min. For optimal reaction time experiments, the same reaction mixture containing 50 mM of PB at pH 7 was incubated at 30 °C for different reaction times.

After the enzyme reactions, the reaction mixture was mixed with an equal volume of methanol and analyzed using high-performance liquid chromatography (HPLC).

### 3.3. High-Performance Liquid Chromatography Analysis

HPLC was performed with an Agilent^®^ 1100 series HPLC system (Santa Clara, CA, USA) equipped with a gradient pump (Waters 600, Waters, Milford, MA, USA) [19]. The stationary phase was a C18 column (Sharpsil H-C18, 5 μm, 4.6 i.d. × 250 mm, Sharpsil, Bei-jing, China), and the mobile phase was 1% acetic acid in water (A) and methanol (B). The elution condition was a linear gradient from 0 min with 40% B to 20 min with 70% B; an isocratic elution from 20 min to 25 min with 70% B; a linear gradient from 25 min with 70% B to 28 min with 40% B; and an isocratic elution from 28 min to 35 min with 40% B. All elutions were performed at a flow rate of 1 mL/min. The sample volume was 10 µL. The detection condition was set at 254 nm.

### 3.4. Purification of Glycosylation Products

The purification process was the same as in our previous work [18,19,20,21,22] and is described briefly below. A 100 mL reaction mixture containing 25 µg/mL *Bt*GT_16345, 1 mg/mL vitexin, 10 mM UDP-G, 10 mM MgCl_2_, and 50 mM PB at pH 7 was incubated at 30 °C for 30 min. After the reaction, an equal volume of methanol was added to stop the reaction. The mixture was then filtered through a 0.2 µm nylon membrane, and the filtrate was injected into a preparative YoungLin HPLC system (YL9100, YL Instrument, Gyeonggi-do, South Korea) equipped with a preparative C18 reversed-phase column (Inertsil, 10 μm, 20.0 i.d. × 250 mm, ODS 3, GL Sciences, Eindhoven, Netherlands) for purification of the biotransformation products. The operational conditions for the preparative HPLC were the same as those used for the analytical HPLC. The eluate corresponding to the metabolite peak in the analytical HPLC was collected, condensed under a vacuum, and then crystallized by lyophilization. Finally, 14.2 mg of compound (**1**) and 15.5 mg of compound (**2**) were obtained, and the structures of the compounds were confirmed, using NMR and mass spectral analysis. The mass analysis was performed on a Finnigan LCQ Duo mass spectrometer (ThermoQuest Corp., San Jose, CA, USA) with electrospray ionization (ESI). ^1^H- and ^13^C-NMR, DEPT, HSQC, HMBC, COSY, and NOESY spectra were recorded on a Bruker AV-700 NMR spectrometer at ambient temperature. Standard pulse sequences and parameters were used for the NMR experiments, and all chemical shifts were reported in parts per million (ppm, *δ*).

*Vitexin-4′-O-β-glucoside* (**1**): yellow powder; ESI/MS *m*/*z*: 593.3 [M − H]^-^, 431.3, 311.3; ^1^H-NMR (DMSO-*d_6_*, 700 MHz): δ H 3.19 (1H, m, H-4′′′), 3.24 (1H, m, H-5′′), 3.27 (1H, m, H-3′′), 3.28 (1H, m, H-2′′′), 3.31 (1H, m, H-3′′′), 3.37 (1H, m, H-5′′′), 3.41 (1H, m, H-4′′), 3.48 (1H, dd, *J*=11.2, 5.6 Hz, H-6′′′a), 3.56 (1H, dd, *J* = 11.2, 4.9 Hz, H-6′′a), 3.69 (1H, d, *J* = 11.2 Hz, H-6′′′b), 3.78 (1H, d, *J* = 11.2 Hz, H-6′′b), 3.81 (1H, t, *J* = 9.8 Hz, H-2′′), 4.69 (1H, d, *J* = 9.8 Hz, H-1′′), 5.03 (1H, *J* = 7.0 Hz, H-1′′′), 6.16 (2H, d, *J* = 8.8 Hz, H-3′, 5′), 6.29 (1H, s, H-6), 6.90 (1H, s, H-3), 8.14 (2H, d, *J* = 8.8 Hz, H-2′, 6′); ^13^C-NMR (DMSO-*d_6_*, 175 MHz): *δ* C 60.5 (C-6′′′), 61.2 (C-6′′),69.5 (C-4′′′), 70.4 (C-4′′), 70.9 (C-2′′), 73.2 (C-2′′′), 73.4 (C-1′′), 76.5 (C-3′′′), 77.1 (C-5′′′), 78.6 (C-3′′), 81,9 (C-5′′), 98.2 (C-6), 100.0 (C-1′′′), 103.5 (C-3), 104.1 (C-10), 104.7 (C-8), 116.6 (C-3′, 5′), 124.5 (C-1′), 128.7 (C-2′, 6′), 156.0 (C-9), 160.3 (C-4′), 160.4 (C-5), 162.7 (C-7), 163.3 (C-2), 182.2 (C-4).

*Vitexin-5-O-β-glucoside* (**2**): yellow powder; ESI/MS *m*/*z*: 593.3 [M − H]^-^, 431.3, 311.1; ^1^H-NMR (DMSO-*d_6_*, 700 MHz): δ H 3.21 (1H, m, H-4′′′), 3.29 (1H, m, H-5′′), 3.31 (1H, m, H-3′′′), 3.35 (1H, m, H-2′′′), 3.39 (1H, m, H-3′′), 3.40 (1H, m, H-4′′), 3.42 (1H, m, H-5′′′), 3.49 (1H, m, H-6′′′a), 3.52 (1H, m, H-6′′a), 3.71 (1H, m, H-6′′′b), 3.72 (1H, m, H-2′′), 3.78 (1H, m, H-6′′b), 4.87 (1H, d, *J* = 9.8 Hz, H-1′′), 4.96 (1H, *J* = 7.7 Hz, H-1′′′), 6.61 (1H, s, H-6), 6.88 (1H, s, H-3), 6.89 (2H, d, *J* = 9.1 Hz, H-3′, 5′), 8.07 (2H, d, *J* = 8.8 Hz, H-2′, 6′); ^13^C-NMR (DMSO-*d_6_*, 175 MHz): *δ* C 60.6 (C-6′′′), 61.3 (C-6′′), 69.4 (C-4′′′), 70.6 (C-4′′), 71.6 (C-2′′), 73.3 (C-1′′), 73.4 (C-2′′′), 75.8 (C-3′′′), 77.1 (C-5′′′), 78.5 (C-3′′), 81,9 (C-5′′), 98.5 (C-6), 101.2 (C-1′′′), 102.6 (C-3), 105.7 (C-10), 107.2 (C-8), 115.9 (C-3′, 5′), 121.2 (C-1′), 129.2 (C-2′, 6′), 154.9 (C-9), 160.8 (C-7), 161.2 (C-5), 161.6 (C-4′), 164.5 (C-2), 182.3 (C-4).

### 3.5. Determination of Aqueous Solubility

The aqueous solubility of vitexin and its glucoside derivatives was examined as follows. Each compound was vortexed in double-deionized H_2_O, PB at pH 6, or pH 8, for 1 h at 25 °C. The mixture was centrifuged at 10,000× *g* for 30 min at 25 °C. The supernatant was filtered through a 0.2 µm nylon membrane, mixed with an equal volume of methanol, and analyzed by HPLC. The concentration of the tested compounds was determined on the basis of their peak areas, using calibration curves prepared by HPLC analyses of authentic samples.

## 4. Conclusions

To our knowledge, this is the first report of vitexin glycosylation and the two produced vitexin glucosides: vitexin-4′-*O*-*β*-glucoside (**1**) and vitexin-5-*O*-*β*-glucoside (**2**). These new compounds were found to have higher aqueous solubility than vitexin. Based on the multiple bioactivities of vitexin, the two new vitexin derivatives have high potential for pharmacological usage in the future.

## Figures and Tables

**Table 1:** Glycosylation activity of the tested GH and GT enzymes toward vitexin.

Enzyme and Class	Name	Source	Conversion (%)
Amylosucrase (GH13)	*Dg*AS	*Deinococcus geothermalis*	N.D. ^1^
Maltogenic amylase (GH13)	*Pg*MA	*Parageobacillus galactosidasius*	N.D.
GT1	*Bs*GT110	*Bacillus subtilis*	5.3% of compound (**1**)
GT1	*Bs*UGT398	*Bacillus subtilis*	13.6% of compound (**1**)
GT1	*Bs*UGT489	*Bacillus subtilis*	6.9% of compound (**1**)
GT28	*Bt*GT_16345	*Bacillus thuringiensis*	17.5% of compound (**1**); 18.6% of compound (**2**)

^1^ N.D. means not detected.

**Table 2:** Aqueous solubility of vitexin and its derivatives at different pH.

Compound	Aqueous Solubility (mg/L)		
	Deionized H_2_O at pH 7	PB at pH 6	PB at pH 8
Vitexin	37.2 ± 2.8 (1) ^1^	7.1 ± 1.2 (0.2)	157.3 ± 3.1 (4.2)
Vitexin-4′-*O*-*β*-glucoside (**1**)	941.4 ± 68.7 (25.3)	943.3 ± 211.6 (25.3)	1802.3 ± 228.6 (48.4)
Vitexin-5-*O*-*β*-glucoside (**2**)	734.6 ± 51.7 (20.0)	444.2 ± 45.0 (11.9)	766.9 ± 21.6 (20.6)

^1^ The fold increase in aqueous solubility of the vitexin glucoside derivatives is expressed as relative to that of vitexin in deionized H_2_O, normalized to 1.

## Data Availability

The data presented in this study are available in the article or supplementary material.

## Supplementary Materials

The following materials are available online. Figure S1. High-performance liquid chromatography (HPLC) analysis of the biotransformation products of vitexin using *Bacillus* GTs. Figure S2. Mass–mass analysis of vitexin-4′-*O*-*β*-glucoside (**1**) in the negative mode. A significant signal at m/z 593.3 corresponded to the molecular weight of vitexin-4′-*O*-*β*-glucoside (**1**) (432 + 180 − 18) in the negative mode [vitexin G − H]^−^. Figure S3. Mass-mass analysis of vitexin-5-*O*-*β*-glucoside (**2**) in the negative mode. A significant signal at m/z 593.3 corresponded to the molecular weight of vitexin-5-*O*-*β*-glucoside (**2**) (432 + 180 − 18) in the negative mode [vitexin G − H]^−^. Figure S4. The ^1^H-NMR (700 MHz) spectrum of vitexin-4′-*O*-*β*-glucoside (**1**) in DMSO-d6. Figure S5. The ^1^C-NMR (175 MHz) spectrum of vitexin-4′-*O*-*β*-glucoside (**1**) in DMSO-d6. Figure S6. The DEPT-135 (175 MHz) spectrum of vitexin-4′-*O*-*β*-glucoside (**1**) in DMSO-d6. Figure S7. The HSQC (700 MHz) spectrum of vitexin-4′-*O*-*β*-glucoside (**1**) in DMSO-d6. Figure S8. The HMBC (700 MHz) spectrum of vitexin-4′-*O*-*β*-glucoside (**1**) in DMSO-d6. Figure S9. The COSY (700 MHz) spectrum of vitexin-4′-*O*-*β*-glucoside (**1**) in DMSO-d6. Figure S10. The NOESY (700 MHz) spectrum of vitexin-5-*O*-*β*-glucoside (**2**) in DMSO-d6. Figure S11. The ^1^H-NMR (700 MHz) spectrum of vitexin-5-*O*-*β*-glucoside (**2**) in DMSO-d6. Figure S12. The ^1^C-NMR (175 MHz) spectrum of vitexin-5-O-*β*-glucoside (**2**) in DMSO-d6. Figure S13. The DEPT-135 (175 MHz) spectrum of vitexin-5-*O*-*β*-glucoside (**2**) in DMSO-d6. Figure S14. The HSQC (700 MHz) spectrum of vitexin-5-*O*-*β*-glucoside (**2**) in DMSO-d6. Figure S15. The HMBC (700 MHz) spectrum of vitexin-5-*O*-*β*-glucoside (**2**) in DMSO-d6. Figure S16. The COSY (700 MHz) spectrum of vitexin-5-*O*-*β*-glucoside (**2**) in DMSO-d6. Figure S17. The NOESY (700 MHz) spectrum of vitexin-5-*O*-*β*-glucoside (**2**) in DMSO-d6.

## Sample Availability

Samples of the compounds are not available from the authors.
