# Supplementary material for: Enzymatic Synthesis of Novel Vitexin Glucosides
Source: Molecules. 2021 Oct 16;26(20):6274. doi: 10.3390/molecules26206274 (PMC8539612; doi:10.3390/molecules26206274)
Supplement: Supplementary file 1 [file molecules-26-06274-s001.zip › molecules-1405085-supplementary.pdf]

Supplemental materials:

Enzymatic Synthesis of Novel Vitexin Glucosides

Jiumn-Yih Wu [1,†], Tzi-Yuan Wang [2,†], Hsiou-Yu Ding [3,†], Yun-Rong Zhang [4], Shu-Yuan Lin [4], and Te-Sheng Chang [4,*]

[1] Department of Food Science, National Quemoy University, Kinmen County 892, Taiwan; wujy@nqu.edu.tw
[2] Biodiversity Research Center, Academia Sinica, Taipei 11529, Taiwan; tziyuan@gmail.com
[3] Department of Cosmetic Science, Chia Nan University of Pharmacy and Science, No. 60, Erh-Jen Rd., Sec. 1, Jen-Te District, Tainan 71710, Taiwan; ding8896@gmail.com
[4] Department of Biological Sciences and Technology, National University of Tainan, Tainan 70005, Taiwan; S10758011@gm2.nutn.edu.tw (Y.-R.Z.); s10758010@gm2.nutn.edu.tw (S.-Y.L.)
* Correspondence: mozyme2001@gmail.com; Tel./Fax: +88-66-260-2137
† These authors contributed equally to this manuscript

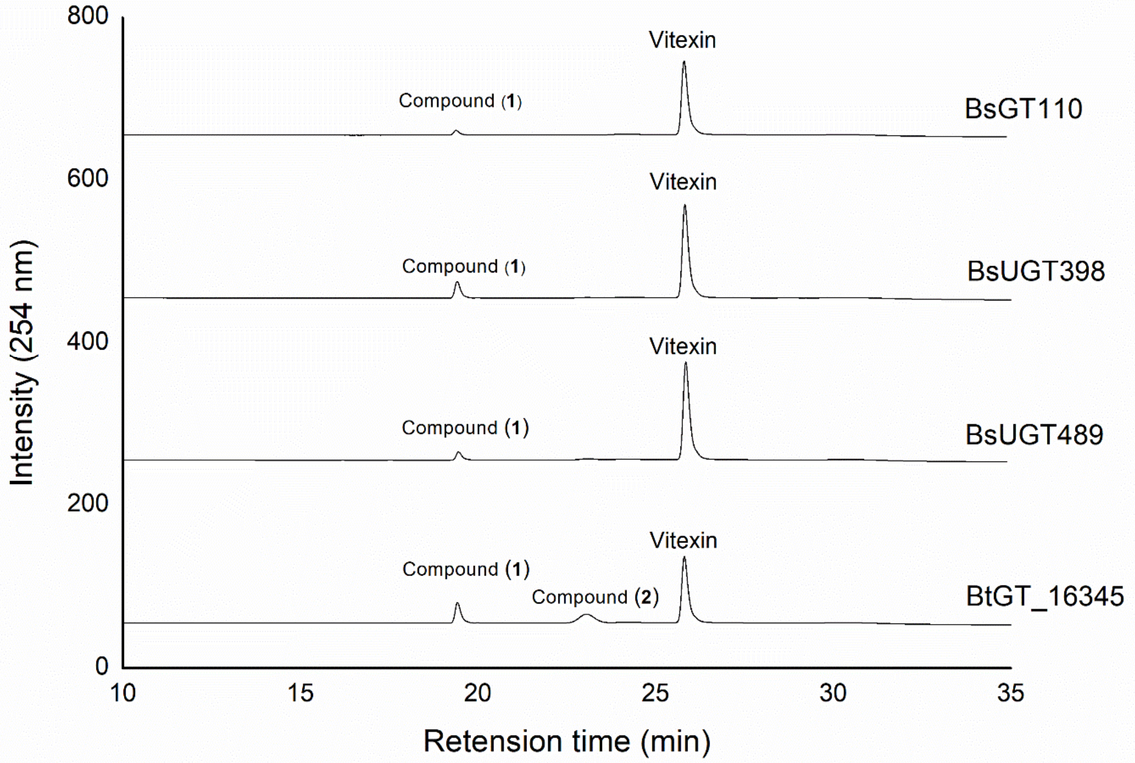

Figure S1. High-performance liquid chromatography (HPLC) analysis of the biotransformation products of vitexin using *Bacillus* GT enzymes. The biotransformation mixture containing 25 µg/mL purified recombinant GT enzymes, 1 mg/mL vitexin, 10 mM uridine diphosphate-glucose (UDP-G), 10 mM $MgCl_2$, and 50 mM phosphate buffer (PB) at pH 6 (BsGT110), pH 7 (BtGT_16345), or Tris buffer at pH 8 (BsUGT398 and BsUGT489) was incubated at 30 °C (BsGT110 and BtGT_16345) or 40 °C (BsUGT398 and BsUGT489) for 30 min. After incubation, the biotransformation products of vitexin by BsUGT398 (a), BsUGT489 (b), BsGT110 (c), and BtGT_16345 (d) were analyzed using HPLC. The HPLC procedure is described in the Materials and methods section.

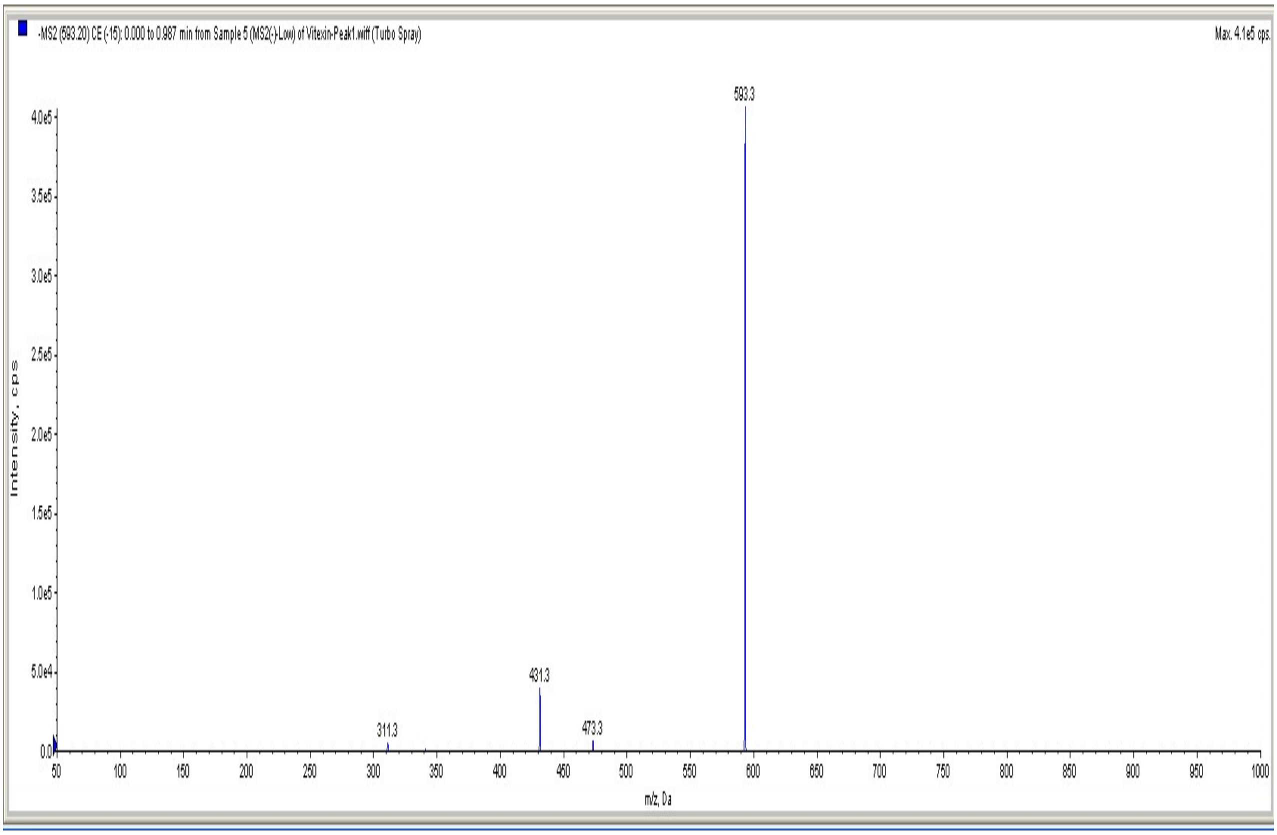

Figure S2. Mass-mass analysis of vitexin-4'-*O*-*β*-glucoside (**1**) at the negative mode. A significant signal at m/z 593.3 showed the corresponding m/z signal of molecular weight 432 of vitexin-4'-*O*-*β*-glucoside (**1**) (432+180-18) at the negative mode vitexin -G – H]$^-$.

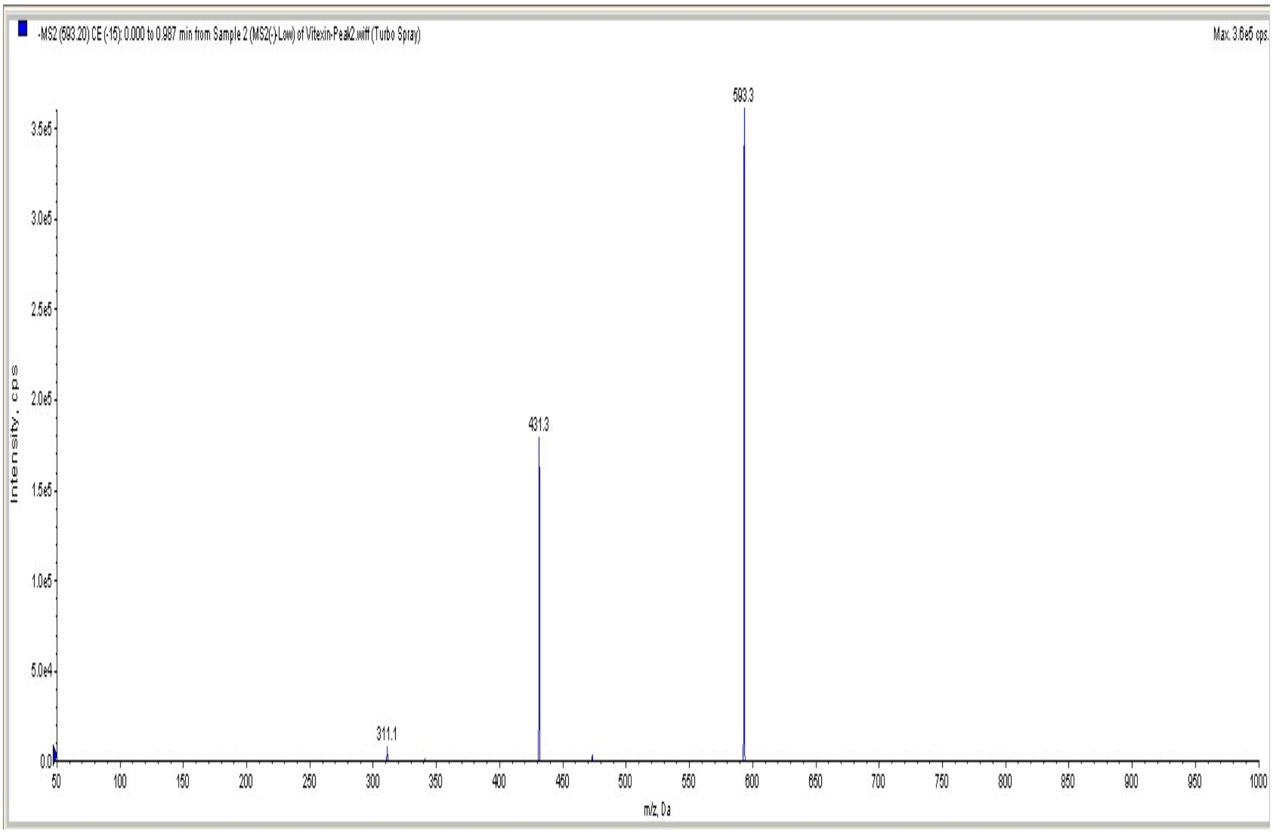

Figure S3. Mass-mass analysis of vitexin-5-*O*-β-glucoside (**2**) at the negative mode. A significant signal at m/z 593.3 showed the corresponding m/z signal of molecular weight 432 of vitexin-5-*O*-β-glucoside (**2**) (432+180-18) at the negative mode [vitexin -G – H]$^-$.

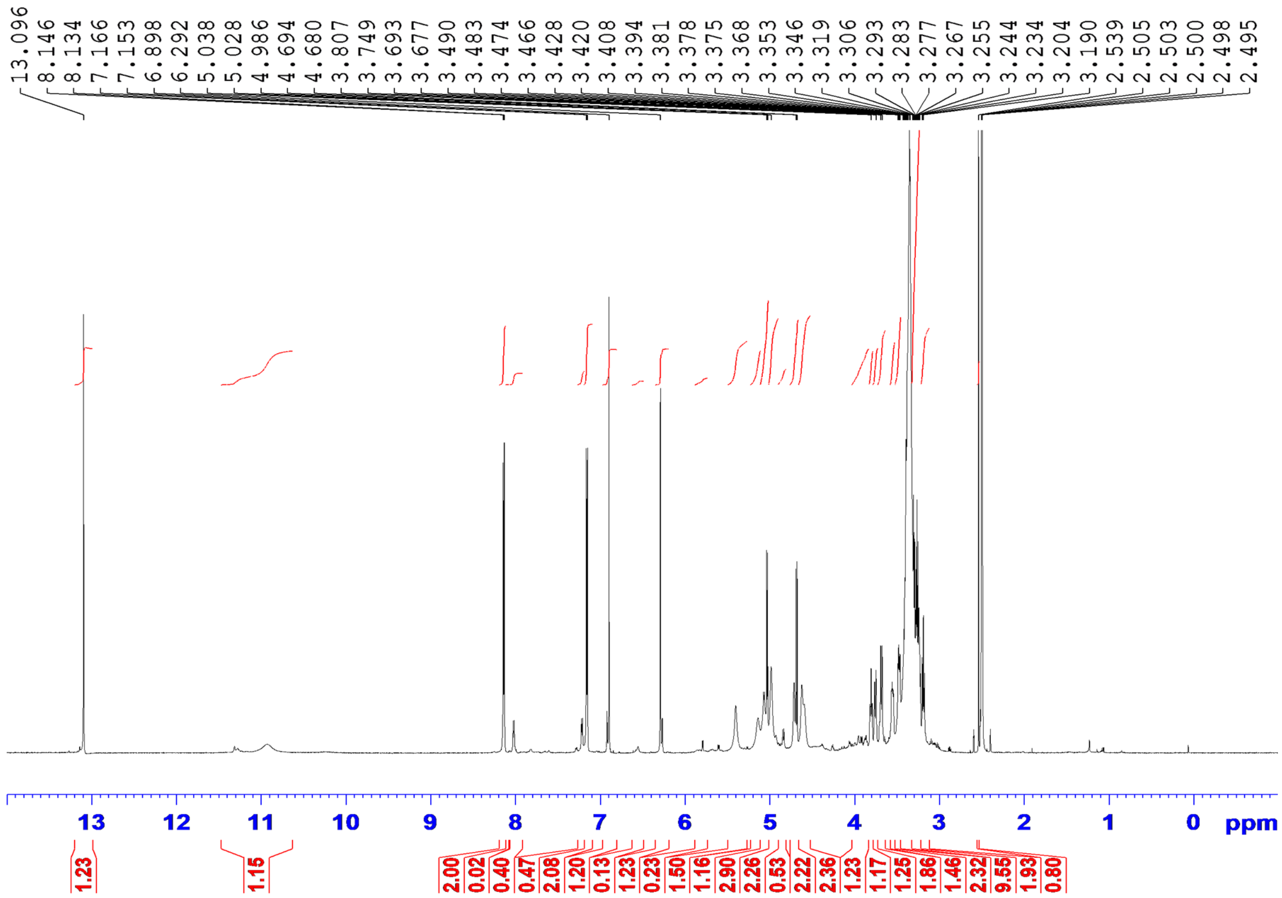

Figure S4. The ^{1}H-NMR (700MHz, DMSO-d_6) spectrum of vitexin-4'-O-β-glucoside (**1**).

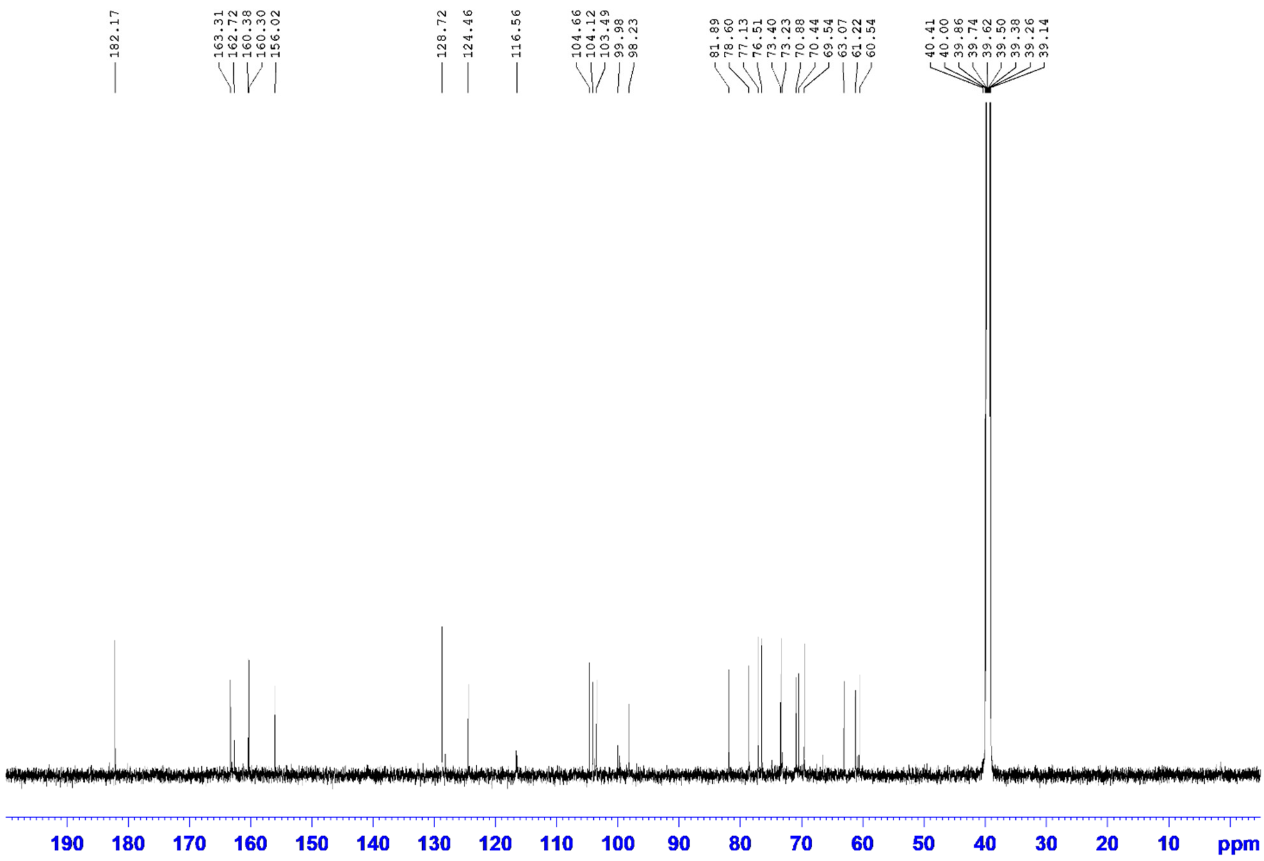

Figure S5. The ^{13}C-NMR (175MHz, DMSO-d_6) spectrum of vitexin-4'-O-β-glucoside (**1**).

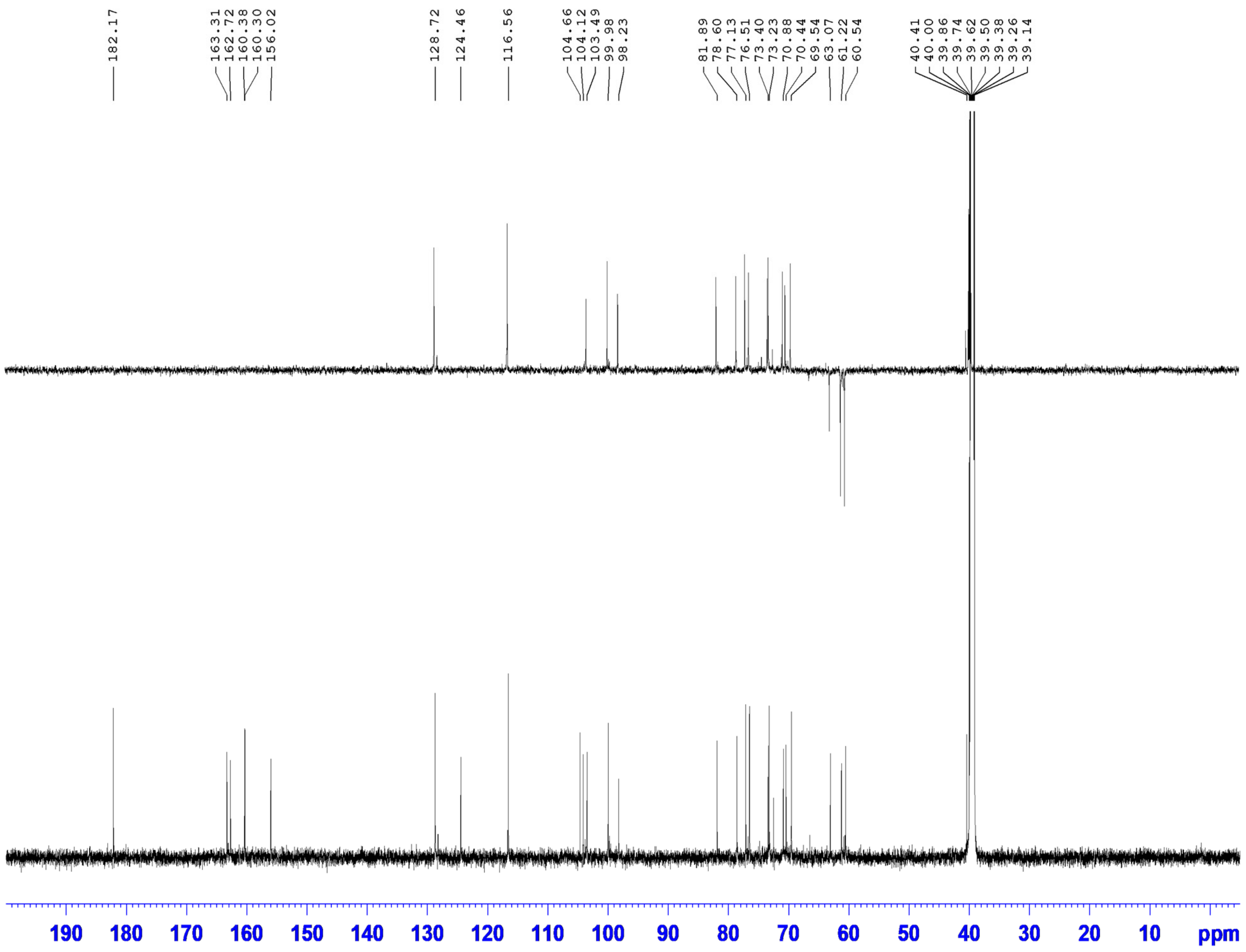

Figure S6. The DEPT-135 (175MHz, DMSO-d_6) spectrum of vitexin-4'-O-β-glucoside (**1**).

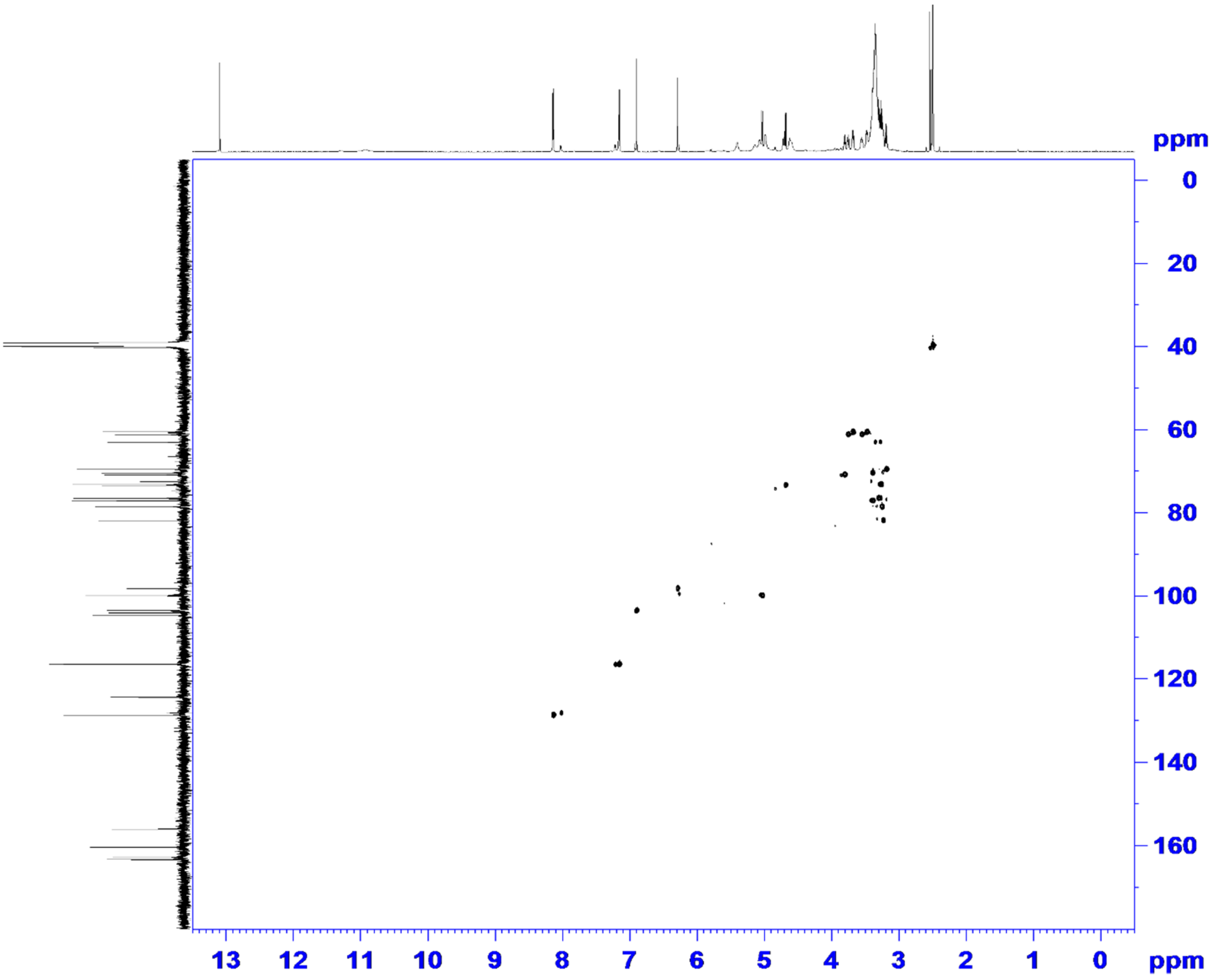

Figure S7. The HSQC (700MHz, DMSO-d_6) spectrum of vitexin-4'-O-β-glucoside (**1**).

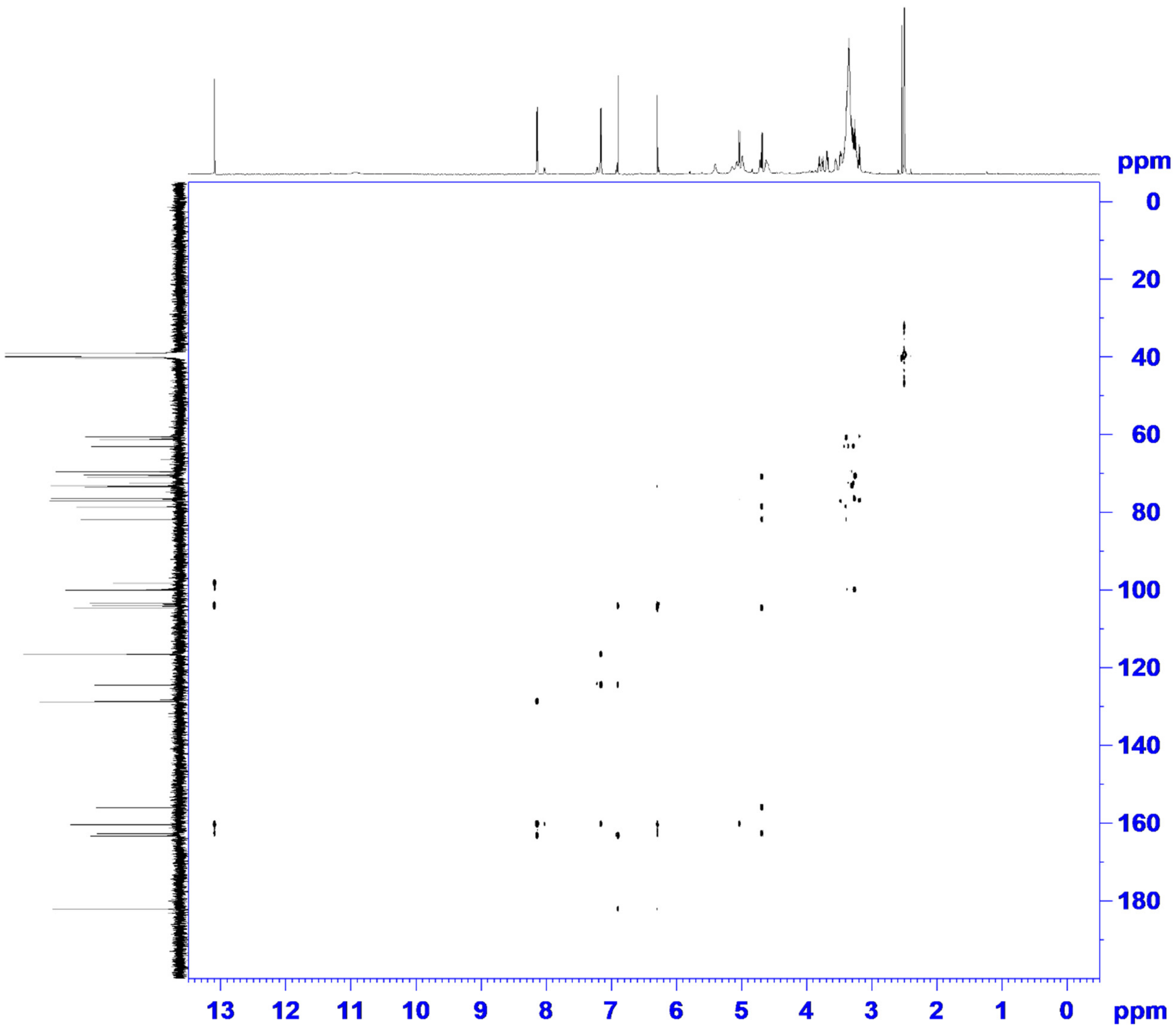

Figure S8. The HMBC (700MHz, DMSO-d_6) spectrum of vitexin-4'-O-β-glucoside (**1**).

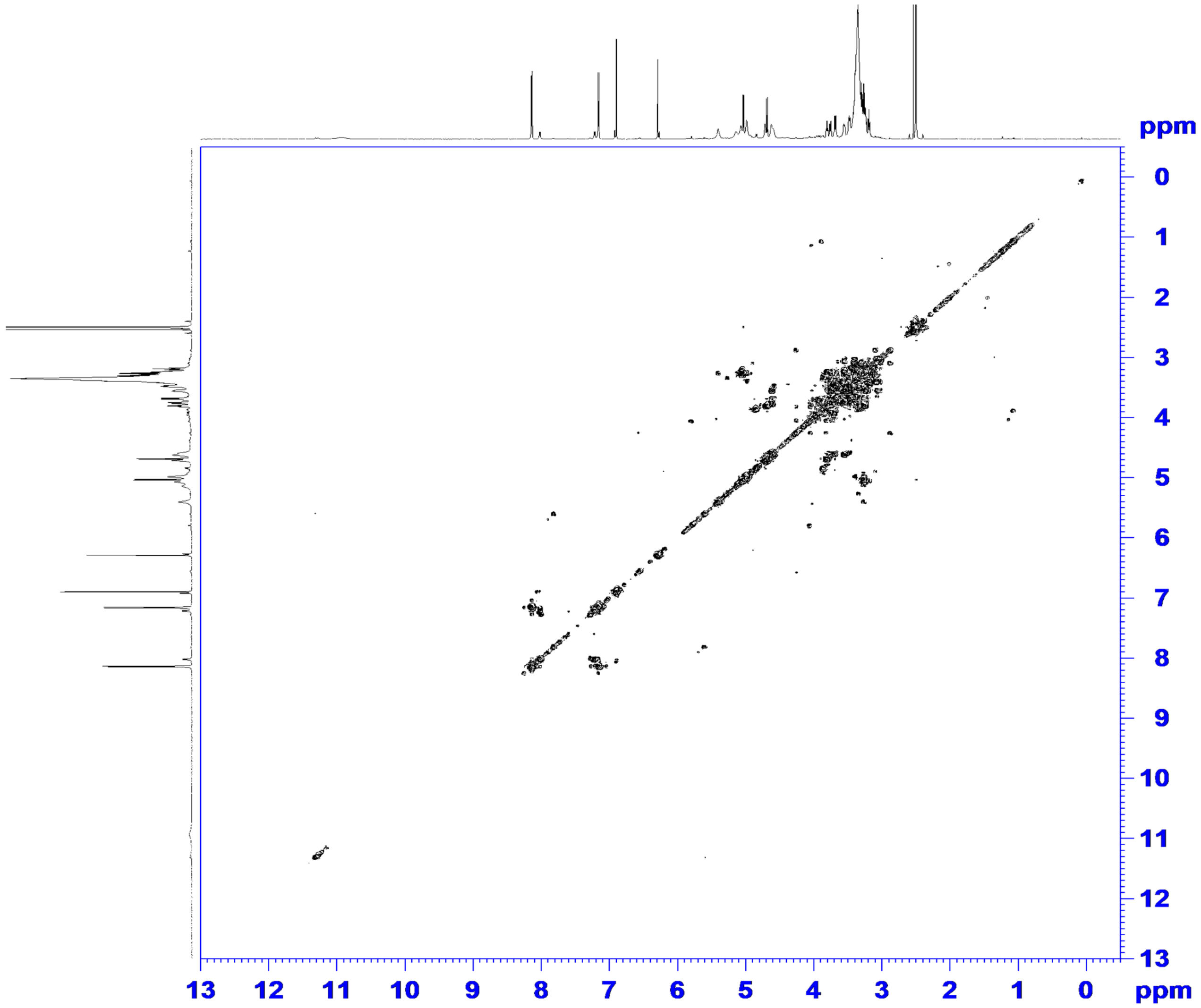

Figure S9. The COSY (700MHz, DMSO-d_6) spectrum of vitexin-4'-O-β-glucoside (**1**).

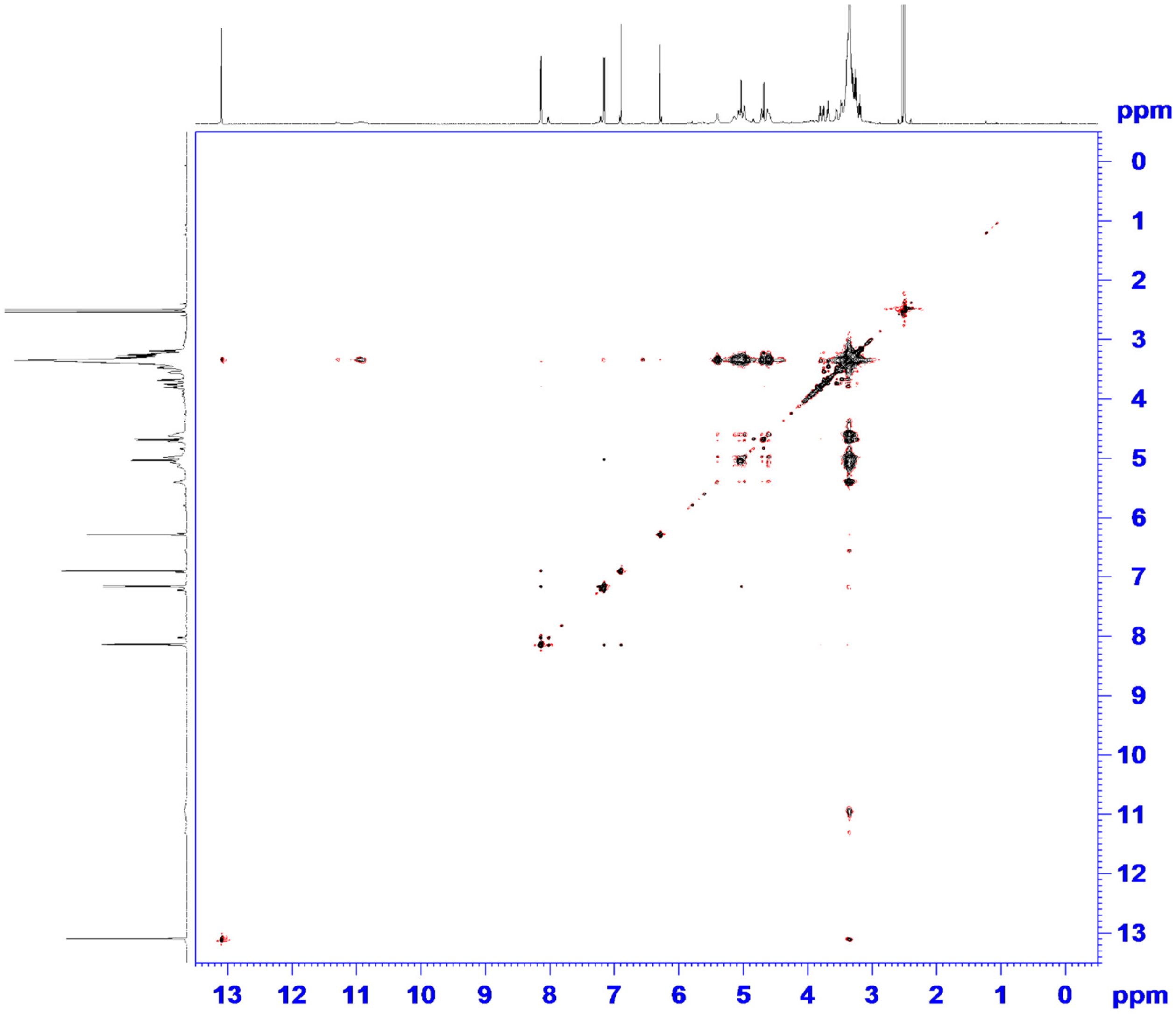

Figure S10. The NOESY (700MHz, DMSO-d_6) spectrum of vitexin-4'-O-β-glucoside (**1**).

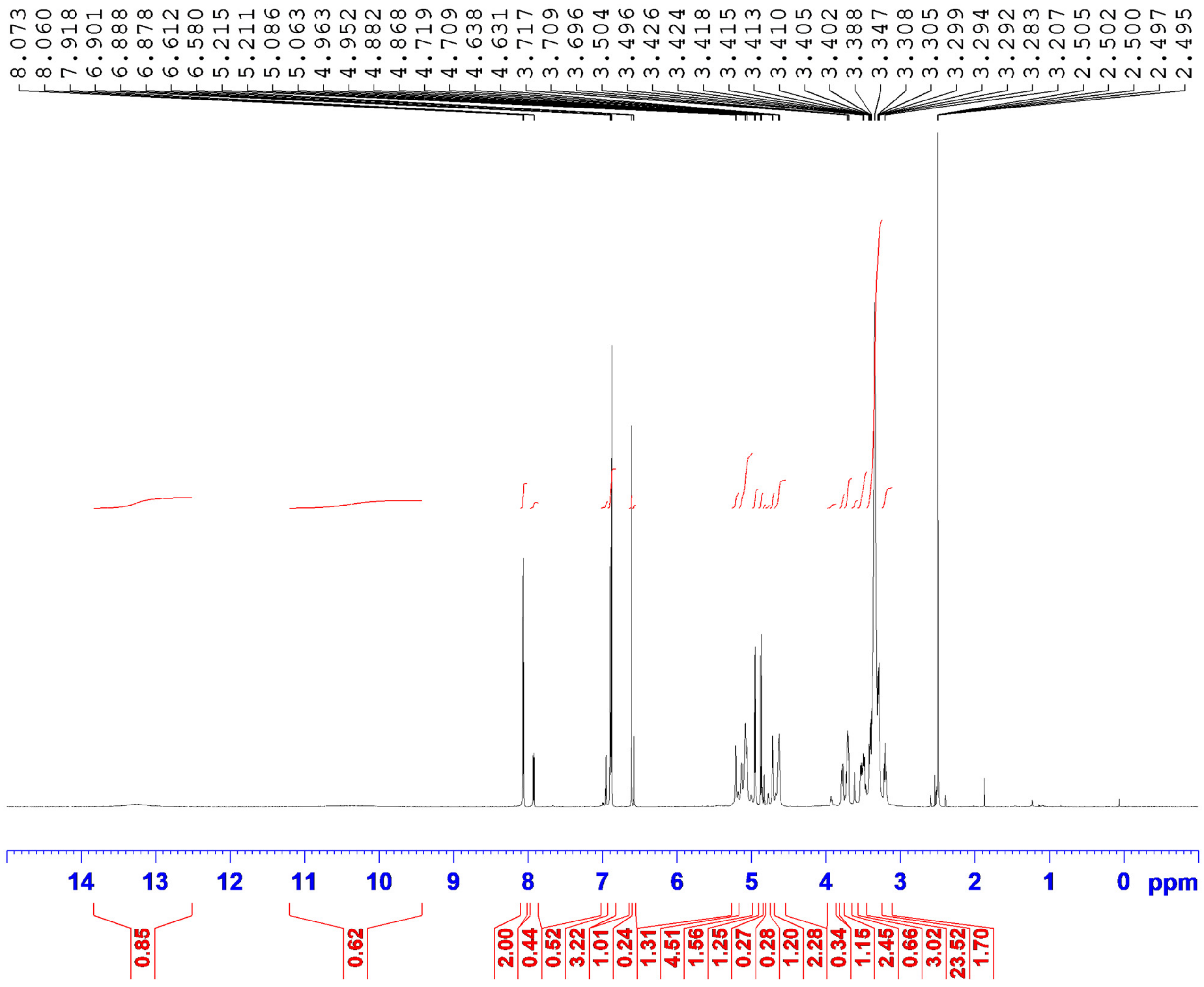

Figure S11. The ^{1}H-NMR (700MHz, DMSO-d_6) spectrum of vitexin-5-O-β-glucoside (**2**).

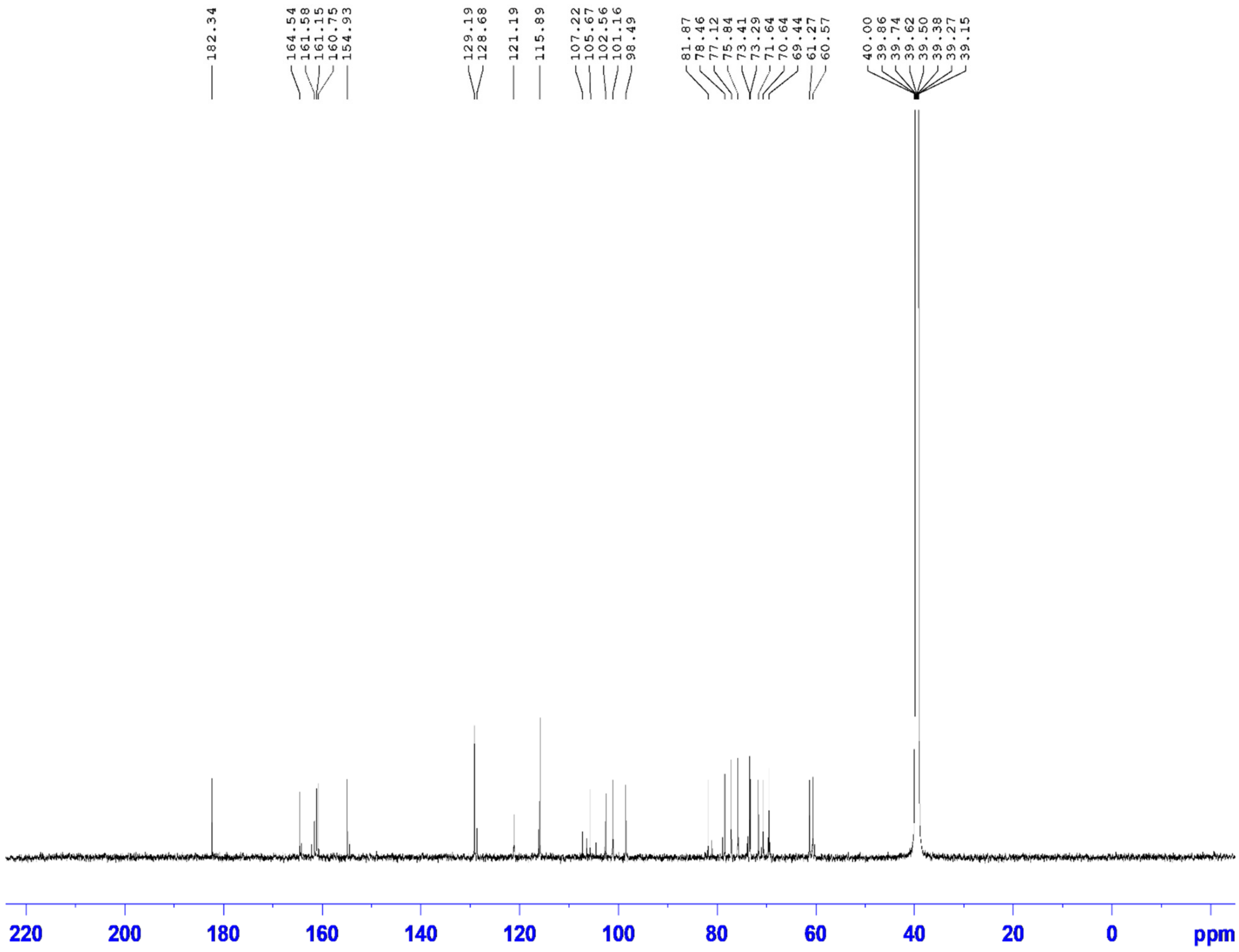

Figure S12. The ^{13}C-NMR (175MHz, DMSO-d_6) spectrum of vitexin-5-O-β-glucoside (**2**).

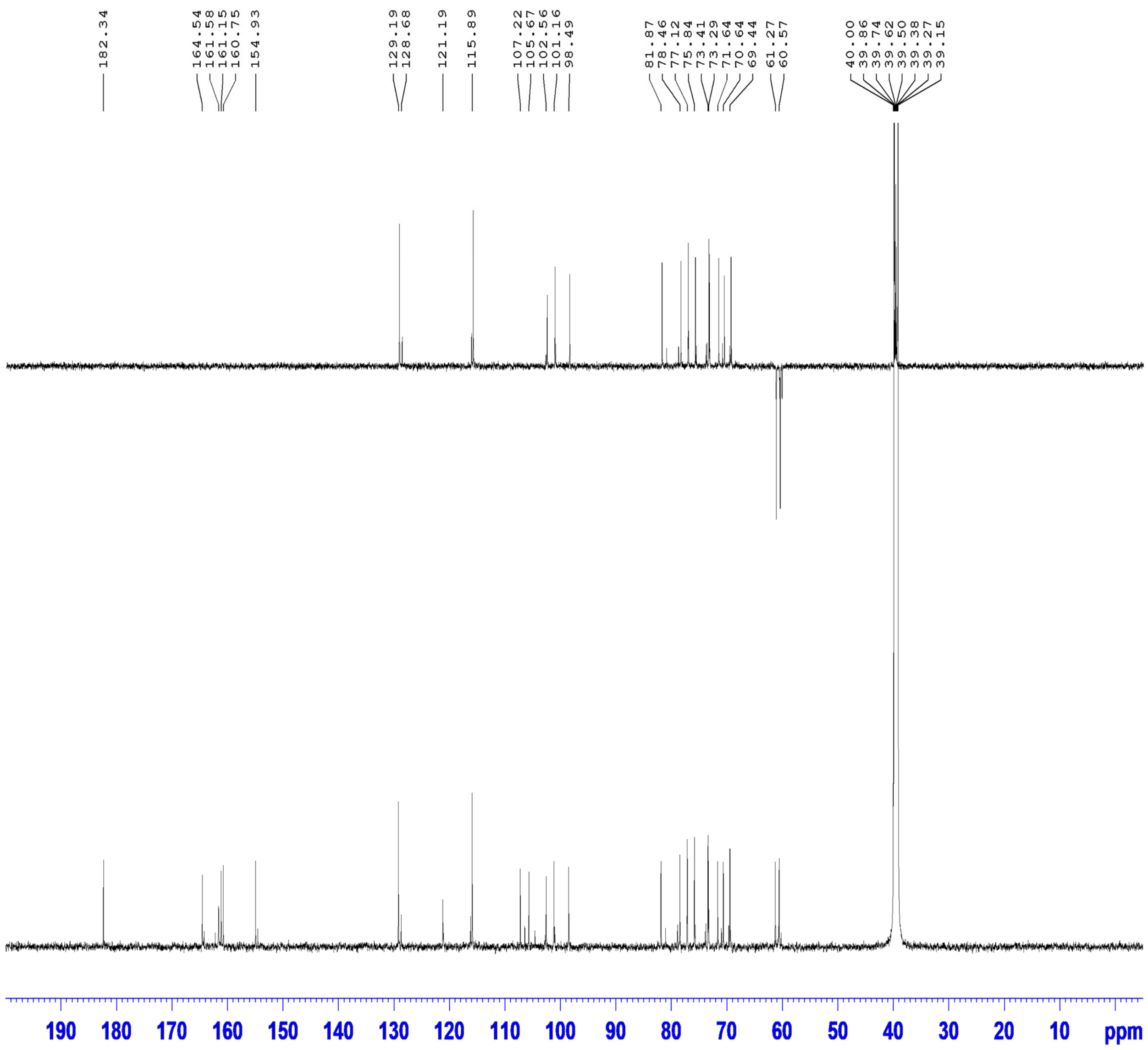

Figure S13. The DEPT-135 (175MHz, DMSO-d_6) spectrum of vitexin-5-O-β-glucoside (**2**).

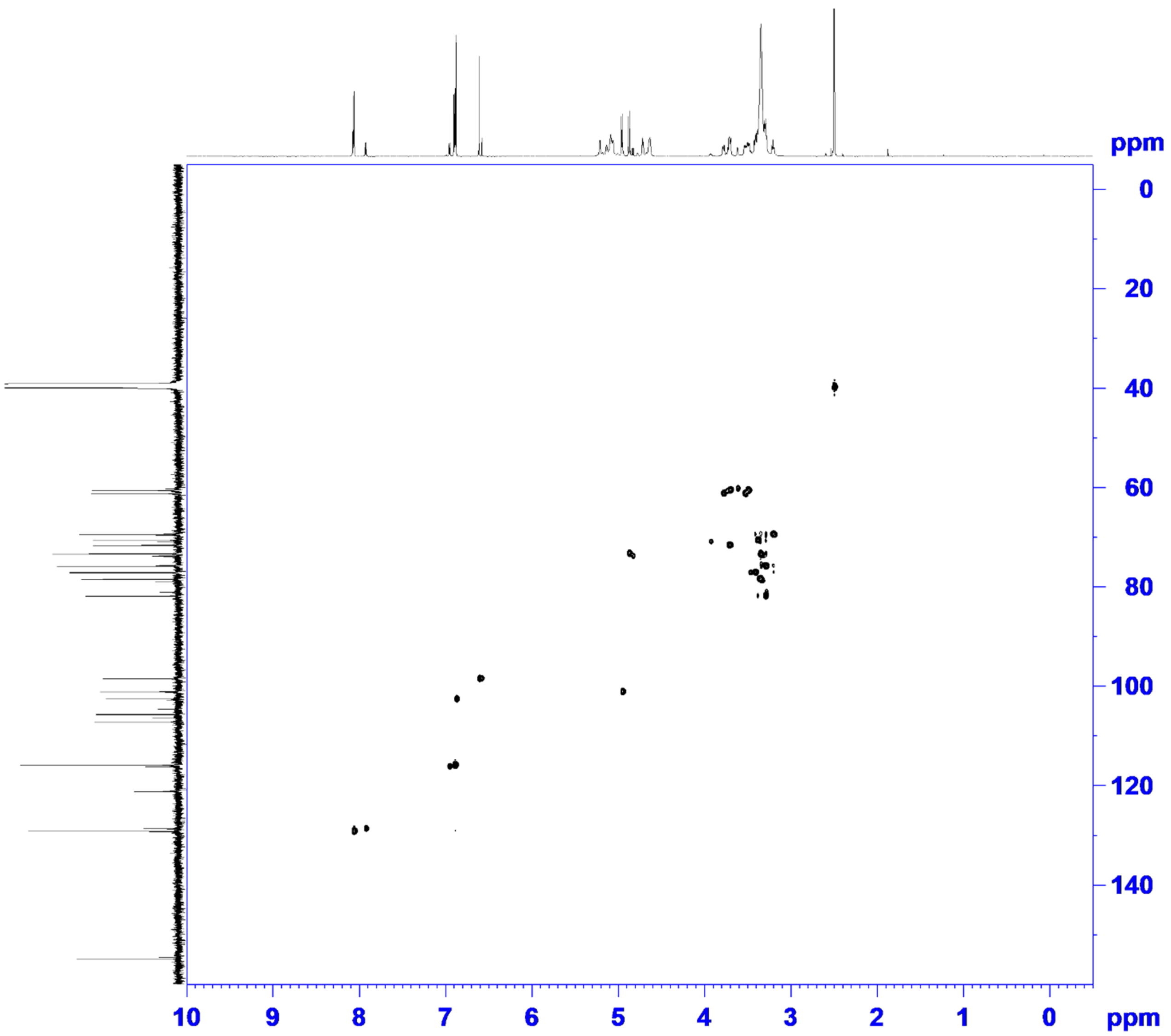

Figure S14. The HSQC (700MHz, DMSO-d_6) spectrum of vitexin-5-O-β-glucoside (**2**).

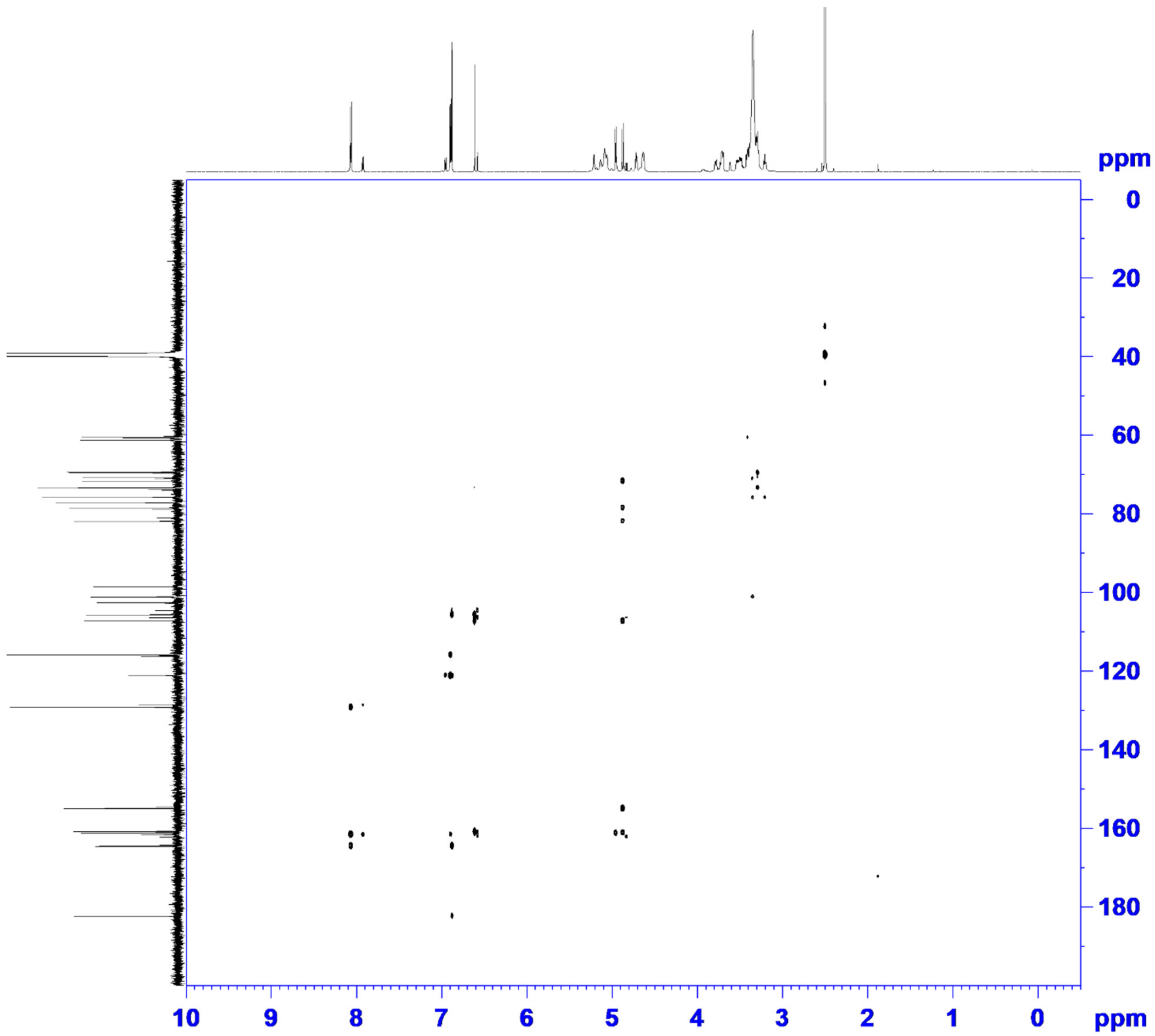

Figure S15. The HMBC (700MHz, DMSO-d_6) spectrum of vitexin-5-O-β-glucoside (**2**).

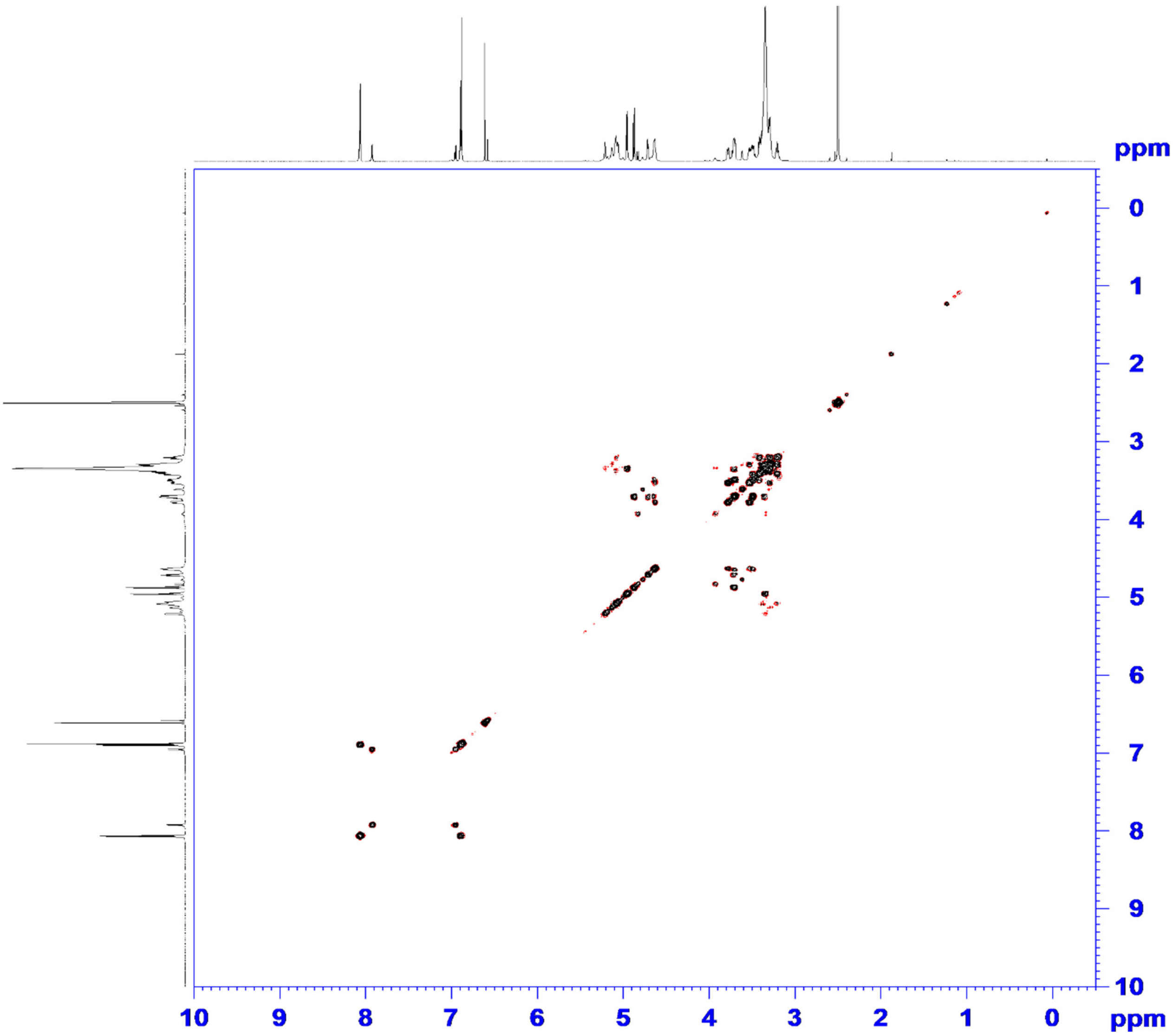

Figure S16. The COSY (700MHz, DMSO-d_6) spectrum of vitexin-5-O-β-glucoside (**2**).

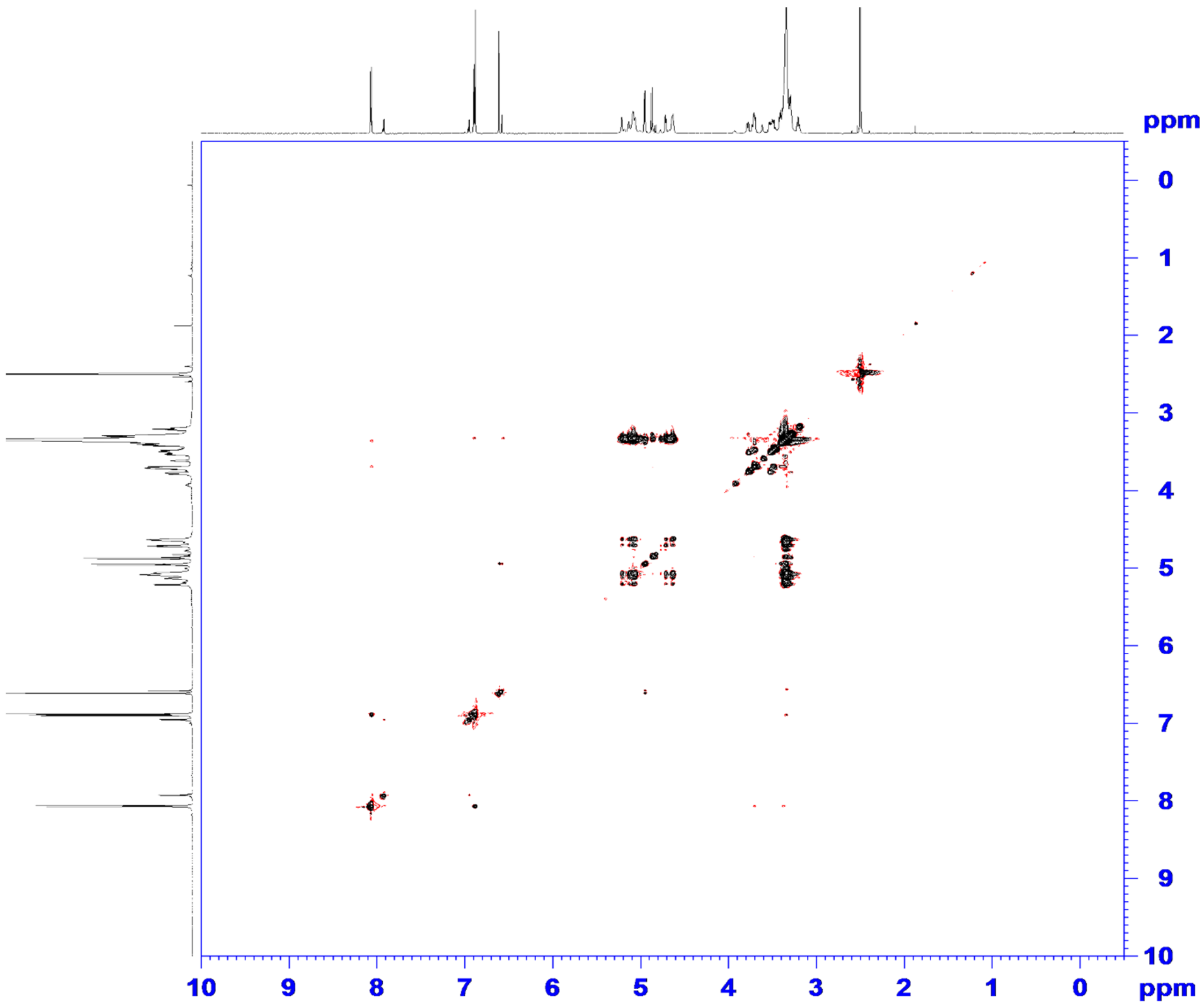

Figure S17. The NOESY (700MHz, DMSO-d_6) spectrum of vitexin-5-O-β-glucoside (**2**).
